# Diffusion in realistic biophysical systems can lead to aliasing effects in diffusion spectrum imaging

**DOI:** 10.1002/mrm.26080

**Published:** 2015-12-30

**Authors:** Luis M. Lacerda, Jonathan I. Sperl, Marion I. Menzel, Tim Sprenger, Gareth J. Barker, Flavio Dell'Acqua

**Affiliations:** ^1^ NATBRAINLAB, Department of Neuroimaging, Institute of Psychiatry, Psychology & NeuroscienceKing's College LondonUnited Kingdom; ^2^ GE Global ResearchMunichBYGermany; ^3^ Department of Neuroimaging, Institute of Psychiatry, Psychology & NeuroscienceKing's College LondonUnited Kingdom

**Keywords:** aliasing, diffusion, DSI, ODF, propagator, resolution

## Abstract

**Purpose:**

Diffusion spectrum imaging (DSI) is an imaging technique that has been successfully applied to resolve white matter crossings in the human brain. However, its accuracy in complex microstructure environments has not been well characterized.

**Theory and Methods:**

Here we have simulated different tissue configurations, sampling schemes, and processing steps to evaluate DSI performances' under realistic biophysical conditions. A novel approach to compute the orientation distribution function (ODF) has also been developed to include biophysical constraints, namely integration ranges compatible with axial fiber diffusivities.

**Results:**

Performed simulations identified several DSI configurations that consistently show aliasing artifacts caused by fast diffusion components for both isotropic diffusion and fiber configurations. The proposed method for ODF computation showed some improvement in reducing such artifacts and improving the ability to resolve crossings, while keeping the quantitative nature of the ODF.

**Conclusion:**

In this study, we identified an important limitation of current DSI implementations, specifically the presence of aliasing due to fast diffusion components like those from pathological tissues, which are not well characterized, and can lead to artifactual fiber reconstructions. To minimize this issue, a new way of computing the ODF was introduced, which removes most of these artifacts and offers improved angular resolution. Magn Reson Med 76:1837–1847, 2016. © 2015 The Authors Magnetic Resonance in Medicine published by Wiley Periodicals, Inc. on behalf of International Society for Magnetic Resonance in Medicine. This is an open access article under the terms of the Creative Commons Attribution License, which permits use, distribution and reproduction in any medium, provided the original work is properly cited.

## INTRODUCTION

Diffusion spectrum imaging (DSI) is a diffusion imaging technique that has been used to explore microstructure and biophysics of living biological systems 1. Based on the q‐space formalism originally introduced by Callaghan et al 2, 3, DSI exploits the direct Fourier relationship between the space of the average spectrum of spin displacements and the MR diffusion‐weighted signal. Succinctly, it is possible to obtain a reconstruction of the ensemble average propagator (EAP), by applying the three‐dimensional (3D) Fourier transform to the diffusion‐weighted signal acquired on a uniform 3D Cartesian grid that covers multiple regularly spaced diffusion encoding directions and diffusion weightings 4.

The diffusion propagator provides unique information about diffusion, allowing a more accurate characterization of the displacement and restriction of water molecules than is possible with traditional diffusion tensor imaging 5. Specifically, it allows measures to be extracted such as return to origin probability (RTO), which reflects the level of restriction of the diffusion environment; mean squared displacement (MSD), which is the average displacement experienced by water molecules in the measured sample; and kurtosis (K), which gives information on how the probability of water molecules displacement deviates from a Gaussian distribution. These indices aid exploration of microstructure both in health and in pathological conditions such as multiple sclerosis (MS) and stroke 6, 7, 8. Moreover, DSI and other model independent methods capable of retrieving the diffusion propagator do not make any assumptions about the underlying biophysical model, making it possible to explore biological domains which are not entirely defined and in which the use of an a priori diffusion model may lead to errors in the interpretation of the underlying biophysics 9.

Despite its potential for providing useful quantitative measures, DSI is a very time consuming technique, requiring sampling schemes often incompatible with a clinical setting 10. Also, to collect measurements at very high q‐values, stronger and longer diffusion gradients are required than for the simpler diffusion tensor imaging (DTI) based approaches, leading to longer echo times and consequently lower signal‐to‐noise ratio (SNR) of the final data 11. To mitigate some of these issues, accelerated methods that explore the intrinsic sparsity of the diffusion propagator, such as compressed sensing and dictionary based‐techniques, have been proposed 12, 13.

But even with the advent of these accelerated techniques, DSI can only use a very limited number of points in each q‐axis (typically 5, 7, or 11 measurements), providing an incomplete description of the true diffusion spectrum. Zero‐padding is often applied to improve the resolution of the propagator though no additional information is added 14. The addition of Hanning windowing to the signal to avoid q‐space truncation artifacts also leads to over smooth profiles both of the EAP and the derived orientation distribution function (ODF).

The ODF is probably the most important output from methods like DSI because it can inform us about the underlying fiber orientation and allow tractography reconstructions in regions of complex white matter (WM) organization 15. An ODF is obtained by radial integration of the propagator, by summing the probability of water molecules displacement along a specific direction. It is extensively used in tractography, where the accurate reconstruction of 3D WM pathways rely on the ODF's ability to resolve multiple fiber orientations within voxels. Until now, however, most of the optimizations for DSI have been tailored specifically for improved angular resolution of the ODF 16. In particular, these studies have focused on using simple fiber models, while more complex tissue configurations and proper exploration of the true underlying propagator have been neglected 17.

This can be critical in particular in clinical or clinical research settings where the presence of pathology may change the diffusion properties of the tissue under investigation. For example, the presence of edema may affect the measures extracted from DSI, and change the nature of the real diffusion propagator, leading to inaccurate quantifications and artifactual fiber reconstructions. In the current study, therefore, we have investigated how biologically plausible changes are reflected in the diffusion propagator and ODFs, as well as the influence of standard processing DSI steps on the final reconstruction. As a result of our observations, we also propose a new method for ODF computation, optimized for a biophysically meaningful range of diffusions within WM. This method is applicable to other model independent techniques and overcomes some of the problems we encounter with traditional DSI processing, namely the process of subtracting the minimum value for all ODF directions and normalizing them by the maximum amplitude (i.e., min–max normalization), which discards the quantitative nature of the ODF.

## THEORY

### DSI Pipeline

In contrast to the simple DTI formalism, q‐space imaging and diffusion propagator formalisms require collecting a large number of points on a regular Cartesian grid, where each point represents a specific direction and diffusion weighting. For single pulsed gradient spin echo (PGSE) sequences, the sensitivity of the measurement to diffusion, and, therefore, the amount of dephasing that the signal undergoes depends on the applied q‐space vector. This quantity is defined by $q=\frac{1}{2\pi }\gamma \delta G$, where γ is the gyromagnetic ratio for the hydrogen nucleus, δ is the duration and G the amplitude of the applied gradients with gradient rise times assumed to be infinitesimal 18. With this formulation, a Fourier relationship can be identified between the measured echo amplitude and the probability of displacement of any spin occurring over the time between application of both gradients, or the diffusion time ( Δ):

(1) $$P(R,\Delta )=\int^{}E\left(q,\Delta \right)e^{-i2\pi q.R}\;dR\;$$

where E refers to the amplitude of the echo divided by the measured signal without diffusion weighting, R consists on the displacement of any spin during the experiment and P(R,Δ) denotes the average propagator, which indicates the probability of such displacements to occur. However, in real DSI experiments, the applied diffusion gradients are of finite duration, and calculating the inverse Fourier transform of the MR signal (Eq. [1]) only leads to an approximate representation of the true average diffusion propagator where the actual molecular spin displacement is underestimated 19. Nevertheless, being able to obtain an estimate of the underlying propagator, or the EAP 4, 14, 20, within each brain voxel still makes DSI and q‐space imaging a very powerful technique to probe the complex microstructural organization in biological tissues.

**Figure 1:**
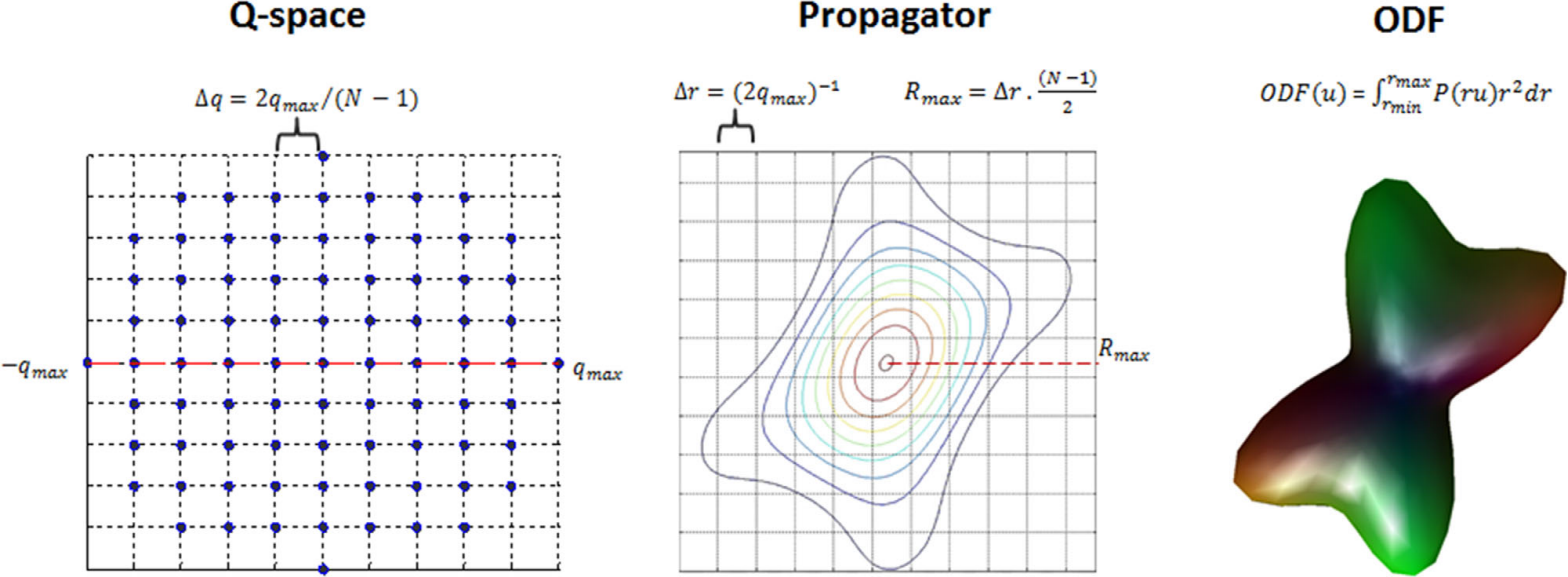
Standard DSI analysis. **Left**: The 3D keyhole Cartesian grid sampling with exclusion of directions in the corners of q‐space grid (2D view presented). **Middle**: Fourier transform of the diffusion signal and reconstruction of the diffusion propagator. **Right**: ODF computation by radial integration of the diffusion propagator.

As shown in Figure 1, because the propagator resolution ( Δr) is inversely proportional to the total q‐space sampling ${\left(2q_{max}\right)}^{-1}$, if very large amplitude gradients (high q‐values) are used, a finer sampling of the displacement space can be achieved (Figure 1, middle panel) 21, 22. At the same time, the field of view of the displacement space that can be explored for a specific type of acquisition is determined by the maximum displacement:

(2) $$\begin{array}{c} R_{max}=\Delta r.\frac{\left(N-1\right)}{2}. \end{array}$$

where N is the number of samples along each axis of our grid. Because of this, it follows that acquisition schemes must be designed to meet the classical Nyquist criteria for data sampling. In particular, the sampling interval Δq (spacing in q between adjacent measurements on the grid) must be sufficient to retrieve the maximum frequency present in the signal 14. Therefore, for a specific acquisition, Δq must be set to at least twice the maximum displacement $R_{max}$ to avoid aliasing.

DSI acquisitions consist on the application of diffusion encoding values within a sphere with radius defined by the maximum q‐value used 16, 23, 24, which has been truncated from a full Cartesian grid. Such truncation gives a speed advantage over full sampling on a rectangular grid, as the corners of q‐space are not acquired, with no significant impact on the estimation of fiber directions following calculation of the ODF 25. Nevertheless, DSI acquisitions remain challenging as the presence of multiple b‐values and use of high amplitude gradients makes it difficult also to run traditional eddy current corrections and at the same time to apply cardiac or peripheral gating acquisitions due to scan time limitations. In addition to acquisition choices, the processing of the diffusion‐weighted data will also affect the final reconstruction of the propagator and the ODF. In particular, it is common to apply zero‐padding to the acquired q‐space data before Fourier transformation to interpolate the diffusion propagator 26, 27. Also, to minimize discontinuities and reduce truncation artifacts, zero‐padding is often performed in conjunction with some degree of filtering of the raw data, with Hanning windowing being mostly commonly used 28, 29, 30. However, this type of filtering may also introduce excessive smoothing in the EAP profile 31. Finally, the ODF is obtained by radial integration of the propagator 32, according to:

(3) $$ODF(u)=\overset{r_{max}}{\underset{r_{min}}{\int }}P\left(ru\right)r^{2}dr.$$

The computed ODF is usually a discrete implementation of its analytic definition, and results from radial integration along several directions. The ranges for ODF integration must also be considered; to date, integration ranges have typically been defined in a largely empirical manner, with $r_{min}$ and $r_{max}$ set to a percentage of the full integration range, with the noise being the main factor considered 14, 33 and no real physical interpretation. Also, to enhance the angular information of the ODF profile, a min–max normalization is usually performed with the minimum ODF value subtracted from all ODF directions and all amplitudes normalized to the maximum ODF value. However, this operation results in an important loss of quantitative information as each ODF profile is rescaled to the same maximum amplitude and, therefore, ODF amplitudes cannot be compared across brain regions or subjects to investigate, e.g., changes in diffusivity along different fiber directions 34.

## METHODS

### Numerical Simulations

The main purpose of our first investigation was to quantify how stable DSI reconstructions are, when changing the nature of the biophysical system involved. The first set of experiments consisted of numerical simulations of two simple biophysical systems, namely an isotropic medium and a single fiber configuration, both consistent with a model of Gaussian diffusion. For both simulations, data were generated with a fix diffusion gradient separation (Δ = 55 ms) and three different realistic acquisition schemes: a “state of the art” (connectome‐like scanner) DSI acquisition defined over a 15 × 15 × 15 Cartesian grid with a diffusion encoding obtained by varying the gradient amplitude in equal steps from 0 to a maximum of 300 mT.m^−1^ (yielding a max q‐value of 102.19 mm^−1^ or maximum b‐value of 21000 s.mm^−2^) and a δ = 8 ms; a “high resolution” DSI acquisition with maximum gradient amplitude of 100 mT.m^−1^ (consistent with a max q‐value of 63.87 mm^−1^ or maximum b‐value of 8000 s.mm^−2^), 11 × 11 × 11 grid yielding 515 sampling points AND a δ = 15 ms; and a “medium resolution” DSI acquisition with maximum gradient amplitude of 40 mT.m^−1^ (consistent with a max q‐value of 47.69 mm^−1^ OR maximum b‐value of 4000 s.mm^−2^) with a 7 × 7 × 7 grid and 123 sampling points AND a δ = 28 ms. For all datasets, data were generated with and without zero‐padding and interpolated to 35 × 35 × 35 and 63 × 63 × 63 cubes 35. Additionally, to achieve a propagator that could asymptotically resemble the true underlying propagator, an acquisition scheme identical to the “state of the art”, but with a grid size of 63 × 63 × 63 and varying gradient amplitude in equal steps from 0 to a maximum of 300 mT.m^−1^ (yielding a max q‐value of 102.19 mm^−1^ or maximum b‐value of 21,000 s.mm^−2^) was also generated. In the first configuration, the isotropic case, different diffusivities were simulated, ranging from 1.0 × 10^−3^mm^2^.s^−1^ to 3.0 × 10^−3^mm^2^.s^−1^ in 0.5 × 10^−3^ intervals. In the second configuration, the single fiber configuration, a single tensor of constant mean diffusivity 36 of 0.7 × 10^−3^mm^2^.s^−1^ was simulated with axial diffusivities of [1.1, 1.3, 1.5, 1.7, 1.9] × 10^−3^mm^2^.s^−1^. For each dataset, the propagator was estimated (with and without Hanning filtering and the ODF was derived by radial integration along 10,832 directions defined on the unit sphere. This number was chosen to give higher angular resolution and better quality in ODF visualization than a more traditional lower number of directions to minimize the likelihood of misattributing visualization issues as fundamental acquisition or processing effects. The estimated ODFs for the isotropic component were then evaluated along 180 directions around a single axis, and the amplitude for each direction extracted. This procedure was repeated for all the three Cartesian grid axes. Similar analysis was repeated for the single fiber system where the fiber was rotated from 0° to 180° relative to the main Z‐axis of the DSI grid, and its local maxima extracted.

### ODF Computation as a Band‐Pass Filter

**Figure 2:**
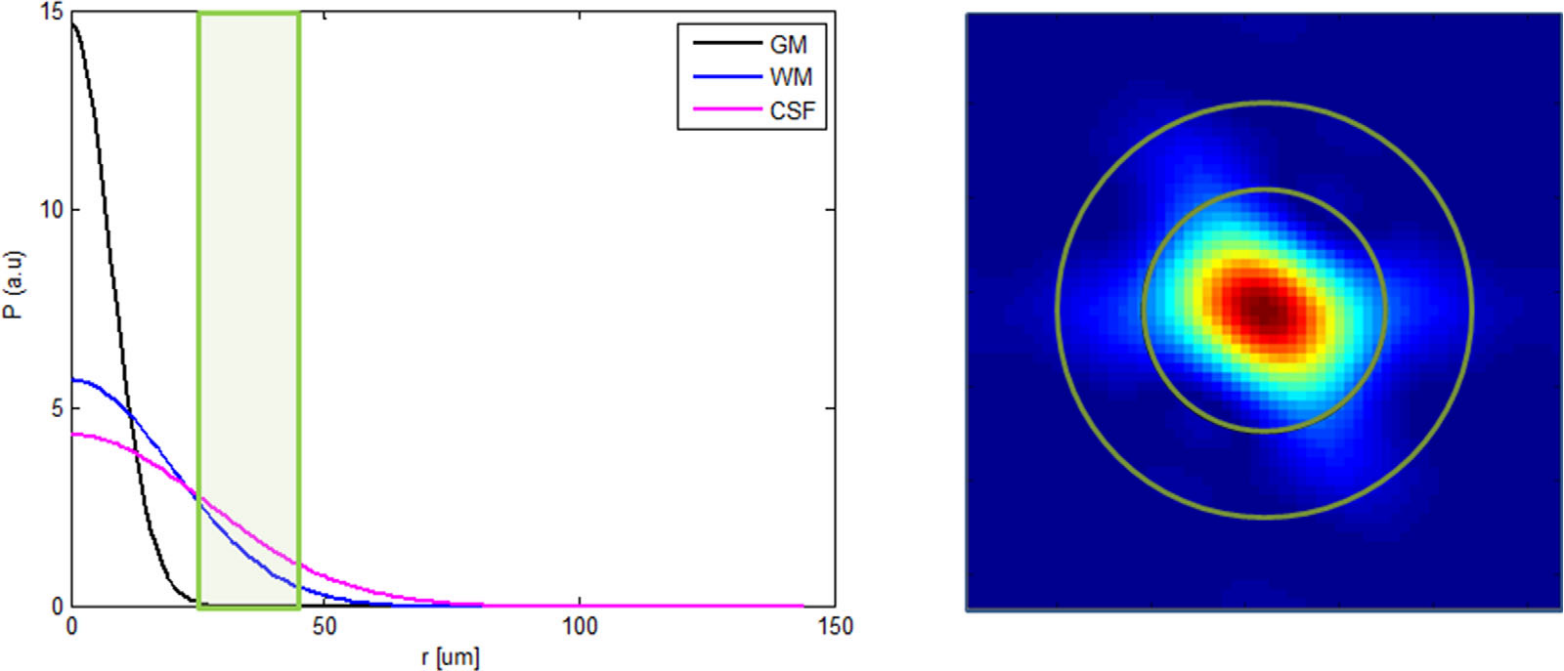
**Left**: The 1D Propagator for specific diffusivities species: GM, WM, and CSF, with 0.7 × 10^−3^ mm^2^.s^−1^, 1.7 × 10^−3^ mm^2^.s^−1^ and 3 × 10^−3^m^2^.s^−1^, respectively, and an example band of the diffusivities of interest. **Right**: Pictorial representation of band pass filter, band which is selected based on particular diffusivity values. Only the probabilities present between the two green circles are of interest for the reconstruction of the ODF (example on the left), for a propagator derived from a 60 degree and a high resolution DSI acquisition, *δ* = 15 ms, Δ = 55 ms.

In a second experiment, we investigated the effect of restricting the range of integration of the ODF calculation as described in Eq. (3) based on the assumption that the mean squared displacement related to fiber orientations will be close to typical values of “axial diffusivity” as measured within single fiber voxels. As shown in Figure 2, a lower bound α and an upper bound β were defined, both representing distinct physical displacements that can be chosen specifically according to the biophysical characteristics of the system under investigation (Eq. (4)).

(4) $$ODF(u)=\overset{\beta }{\underset{\alpha }{\int }}P\left(ru\right)r^{2}\;dr.$$

This displacement is given by $R=\sqrt{6\;D\;t_{diff}}$, where D is the diffusivity of the tissue, and *t*_*diff*_ is the diffusion time (Fig. 2). By restricting the integration range, we can then use the ODF calculation as a sort of band‐pass filter to recover information only related to the diffusion characteristics of WM, excluding contribution from gray matter (GM) and cerebrospinal fluid (CSF)/edema and improving angular resolution. To test our hypothesis, a more complex system was simulated with two fibers crossing at 0°, 15°, 30°, 45°, 60°, 75°, and 90°, modeled as the sum of two fibers with the same diffusivity profile of [1.7 0.2 0.2] × 10^−3^ mm^2^.s^−1^ and no diffusional exchange. The diffusion propagator was calculated with and without partial volume contamination (with GM and CSF), only for the “high resolution” DSI scheme, and again the effects of Zero‐padding and Hanning Filtering were investigated. Additionally only the values above a threshold of 5% in the propagator were kept, as a mean to help decrease the contribution of spurious peaks in the ODF reconstruction. Band‐pass filtered ODFs were generated with different combinations of *α* and *β*, varying the amount of displacement from GM ( $R_{GM}$) and WM ( $R_{WM}$) to be integrated (Fig. 2). These values were chosen to ensure that most of the GM signal has already decayed while covering the range over which significant WM signal remains, with $D_{GM}=0.7\;\times \;10^{-3}{\text{mm}}^{2}.s^{-1}\;$ and $D_{WM}=1.7\;\times \;10^{-3}{\text{mm}}^{2}.s^{-1}$ 36, 37. To compare the band‐pass filtered ODFs with standard processing, ODFs without any integration restrictions and with min‐max normalization were also computed.

### Human In Vivo Data

To validate the results of the numerical simulations, DSI was acquired of a normal adult human subject. To reduce scan time, coverage was restricted to a region expected to demonstrate partial volume contamination, namely the corpus callosum and ventricles. Data acquisition was performed using a 3 Tesla (T) GE MR750 clinical MR scanner with a maximum gradient strength of 50 mT.m^−1^ and slew rate of 200 mT.m^−1^.ms^−1^ and a 32‐channel head coil. Images were acquired using a single‐shot echo‐planar imaging sequence;  δ = 32 ms,  Δ = 55 ms; max q‐value of 68.12 mm^−1^ and b‐value of 8000 s.mm^−2^; echo time of 116.8 ms; repetition time 1700 ms; matrix size = 96 × 96; 11 slices; isotropic voxel size 2.5 × 2.5 × 2.5 mm^3^; four repetitions of the complete protocol were collected for signal averaging purposes. For each voxel, q‐space was sampled on Cartesian grid points within a 3D sphere with diameter of 11, i.e., 11 × 11 × 11 grid, yielding 514 diffusion‐encoding directions. Additionally, 18 b0 images were acquired interspersed and used for motion correction. Total acquisition time was approximately 60 min. The four repetitions were then averaged before propagator and ODF reconstructions. The reconstruction pipeline was identical to the one adopted for the second set of simulations, where the presence of Hanning filter and restriction in the ODF integration ranges was investigated. To investigate the effect of choosing different grid sizes of the Cartesian grid two under‐sampled datasets with 9 × 9 × 9 and 7 × 7 × 7 grid sizes were generated and different integration ranges were compared.

## RESULTS

**Figure 3:**
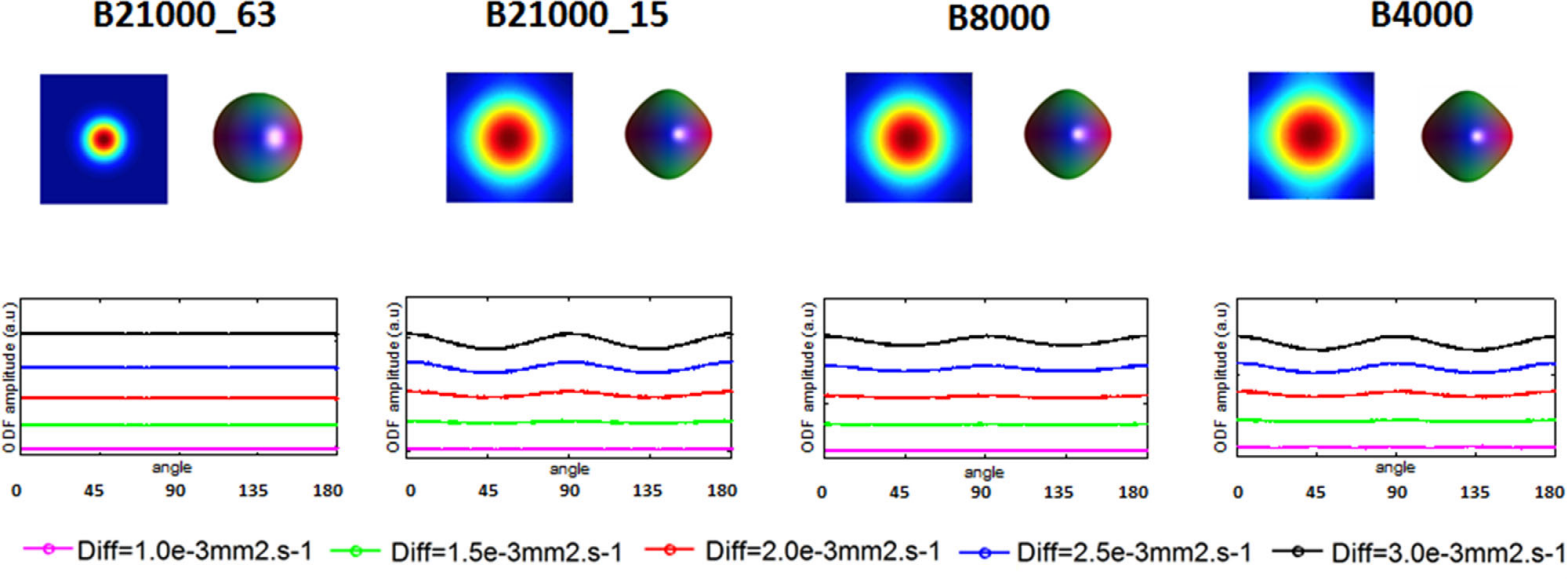
Effect of changing the biophysical system, in an isotropic scenario, and the experimental setup on the amplitude of the ODF. Different isotropic compartments with different diffusivities ranging from 0.5 × 10^−3^ mm^2^.s^−1^ to 3 × 10^−3^ mm^2^.s^−1^ were simulated for an asymptotic, a state of the art, a high DSI and a medium DSI schemes. ODFs were derived from radial integration of the diffusion propagator without any Hanning filtering, and its amplitude measured over 180 different angles around a circumference.

The rationale for this study and in particular for our first investigations was not only to explore the stability of DSI reconstructions accordingly to changes in the biophysical system but how those changes are evaluated. Figure 3 depicts changes in the displacement field of the propagator as a result of varying the diffusivity of the simulated isotropic compartment, the applied b‐value and grid size. It is visible from this figure that for lower b‐values and smaller grid sizes, the displacements is not uniform across the field of view of the propagator, and it corresponds to a nonperfectly spherical profile. More in detail, by measuring the amplitude of the ODF, we observe that, with the exception of the “asymptotic” case, its value is not constant for all angles, as we should expect from isotropic diffusion. These effects are more pronounced for fast diffusivity and decrease when the diffusivity of the system is lower. These results suggest that with realistic DSI schemes some fast diffusion components may not be well characterized and lead to aliasing effects. The results reported in this figure display diffusion propagators generated without Hanning filtering to investigate mainly the effect of different acquisition schemes rather than the effect of processing. Similar results were also obtained from data where Hanning filtering was used.

**Figure 4:**
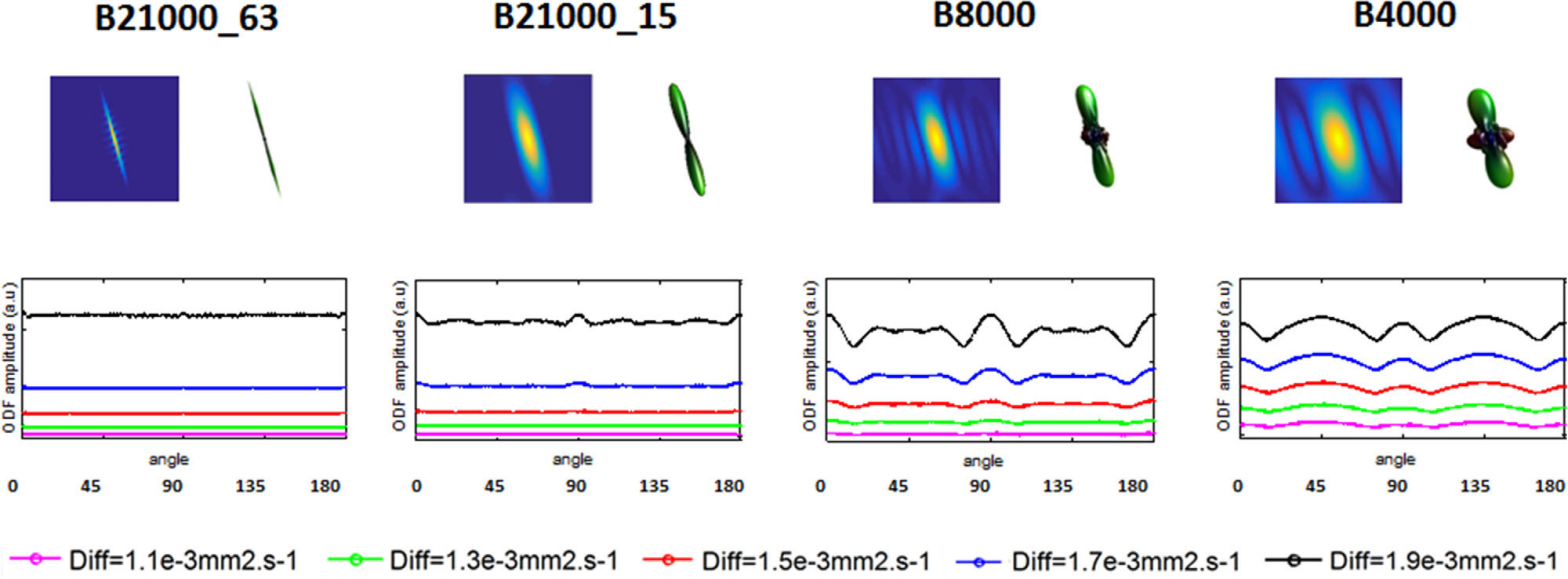
Effect of changing the biophysical system, in a single fiber scenario, and the experimental setup on the amplitude of the ODF. Different single fibers with different diffusivities ranging from 1.1 × 10^−3^ mm^2^.s^−1^ to 2.1 × 10^−3^ mm^2^.s^−1^ were simulated for an asymptotic, a state of the art, a high DSI, and a medium DSI schemes and for 180 different angles. ODFs were derived from radial integration of the diffusion propagator without any Hanning filtering, and its amplitude measured over 180 different angles around a circumference.

Figure 4 shows similar, but more localized, effects in the single fiber case. Whereas in the isotropic scenario a single ODF is evaluated along different angles, here different fibers were simulated for different angles, with the same biophysical properties, and its amplitude measured along the maximum direction, for different acquisition schemes. Again we can see that for progressively lower b‐values the spread of displacements in the propagator is larger and that directly affects the reconstruction of the ODF; these effects are once again even more evident for higher diffusivity values. An example of an ODF and its associated propagator is displayed for a 15‐degree angle and the simulated acquisition schemes.

**Figure 5:**
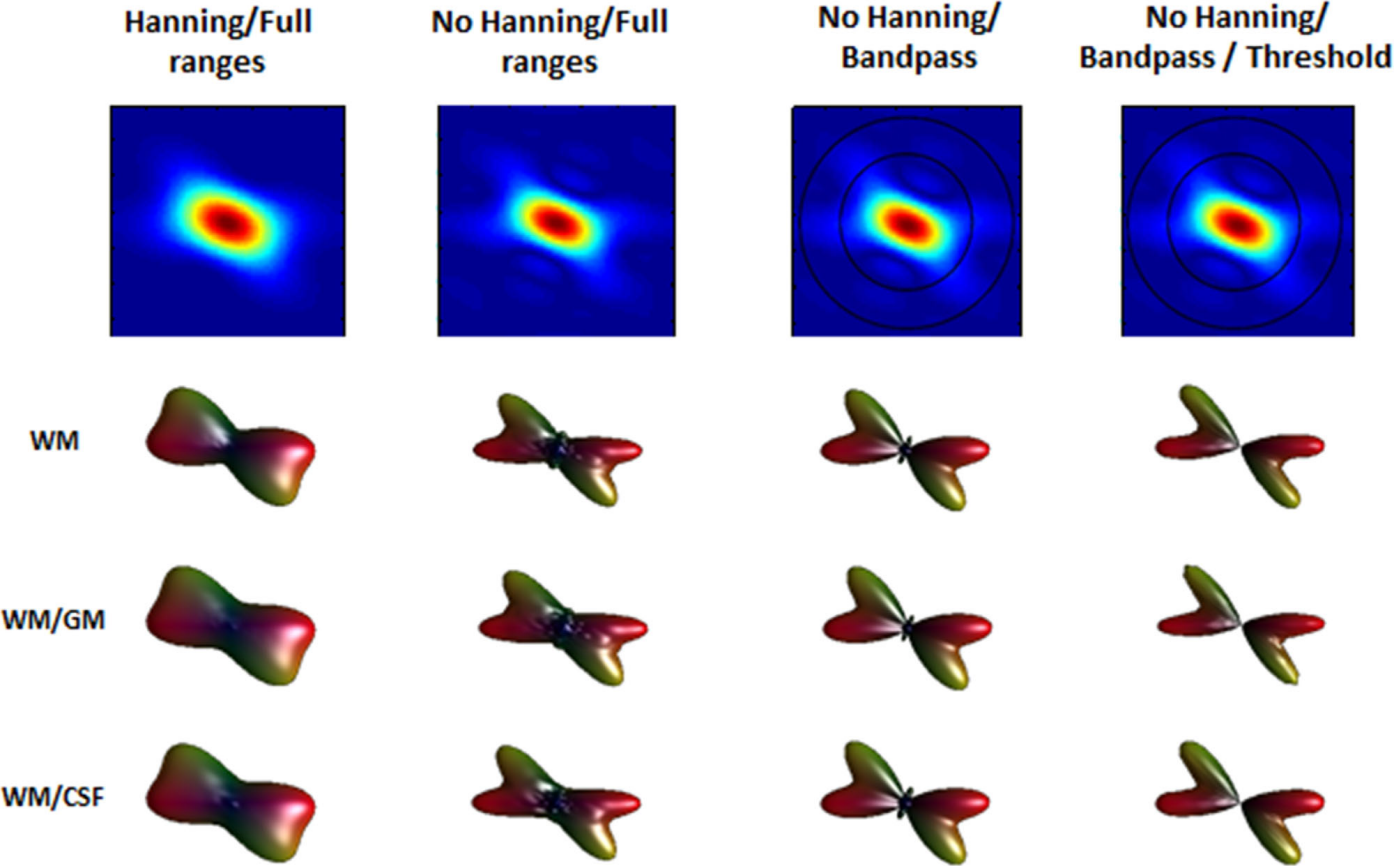
Effect of partial volume contamination and processing steps of the propagator and ODF on a simulated crossing fiber (45 degrees). Fiber diffusivity of 1.9 × 10^−3^ mm^2^.s^−1^, and a high resolution DSI scheme, max b‐value = 8000 s.mm^−2^. From left to right, ODFs generated: with Hanning filtering and full ranges of integration; without Hanning filter and full ranges of integration; without Hanning filter and band‐pass integration approach (α = 2*R_GM_; β = 2.2*R_WM_); without Hanning filter, band‐pass integration approach (α = 2*R_GM_; β = 2.2*R_WM_) and threshold on the propagator. From top to bottom: **WM** only; contamination with GM (25%); contamination with CSF (25%).

The influence of partial volume contaminations and the effect of changing the processing of diffusion propagator and ODFs reconstruction are shown in Figure 5.

In the first two columns, the propagator was generated with and without Hanning filter and the ODF reconstructed without restriction in the integration ranges. The third and fourth column display ODFs reconstructed with the band‐pass approach, the latter including an additional threshold on the propagator values as described in the methods section. Furthermore, the effect of partial volume contamination with GM and CSF (both 25%) is displayed. It is easily seen that the Hanning filtering reduces the artifacts present in the diffusion propagator at the expense, however, of a lower angular resolution of the ODF, for all scenarios. The effect of restricting the integration ranges is important for reduction of some of the artifacts due to partial volume contamination and also for an increased angular resolution of the ODF. Additionally, the use of a threshold in the diffusion propagator appears to be beneficial as ringing artifacts remaining after the application of the band‐pass are further removed.

**Figure 6:**
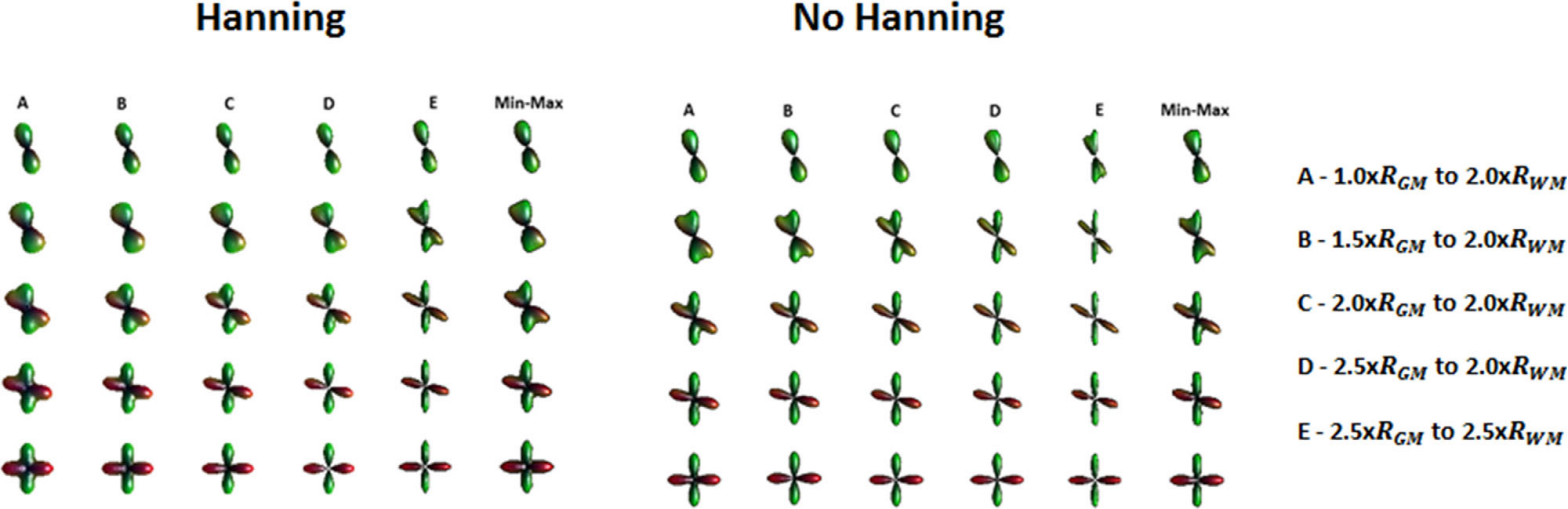
Comparison between standard ODF computation with min–max normalization (first column) to the “band pass” processing in which ODFs are computed with different integration ranges: A ‐ 1.0 × $R_{\text{GM}}\;$ to 2.0 × $R_{\text{WM}}$; B ‐ 1.5 × $R_{\text{GM}}\;$ to 2.0 × $R_{\text{WM}}$; C ‐ 2.0 × $R_{\text{GM}}\;$ to 2.0 × $R_{\text{WM}}$; D ‐ 2.5 × $R_{\text{GM}}\;$ to 2.0 × $R_{\text{WM}}$; E ‐ 2.5 × $R_{\text{GM}}\;$ to 2.5 × $R_{\text{WM}}$.

Additional simulations were then performed to identify the best pair of integration ranges to be used and results are summarized in Figure 6. As comparison, a min–max normalized ODF computed without restricting the integration ranges is also shown. The influence of Hanning filtering in the final results was also investigated and the angular errors (AE) with the ground truth computed for all ODFs (Tables 1 and 2).

**Table 1:** Angular Error between Simulated Ground Truth and Reconstructed ODF Crossing Angle, for Different Integration Ranges and Min–Max ODFs, Generated with Hanning

	Hanning					
	A	B	C	D	E	Full
30°						
45°				6.40	5.65	
60°	10.65	9.60	7.13	4.21	2.03	2.25
75°	4.87	3.68	1.98	0.56	0.44	1.71
90°	0.00	0.00	0.00	0.00	0.00	0.00

**Table 2:** Angular Error between Simulated Ground Truth and Reconstructed ODF Crossing Angle, for Different Integration Ranges and Min–Max ODFs, Generated without Hanning

	No Hanning					
	A	B	C	D	E	Full
30°					4.64	
45°	5.52	4.89	2.35	1.02	1.50	0.99
60°	2.70	0.68	1.13	1.79	1.07	0.89
75°	0.61	0.22	0.81	0.76	0.24	0.73
90°	0.00	0.00	0.00	0.00	0.00	0.00

Tables 1 and 2 present a more quantitative analysis of the improvement in angular resolution from the use of adequate integration ranges in the ODF reconstruction. The integration range that offers better angular resolution by giving a lower angular error, is the one that corresponds to $\alpha =2.5*R_{GM}\;\text{and}\;\beta =\;2.5*R_{WM}$, for both the case with and without Hanning filtering. It is also important to note, that when compared with the standard min–max ODF, the band pass approach was able to resolve crossings down to 30 degrees, as opposed to 35 degrees, for the non‐Hanning scenario, and 40 degrees instead of 45, for the Hanning configuration.

**Figure 7:**
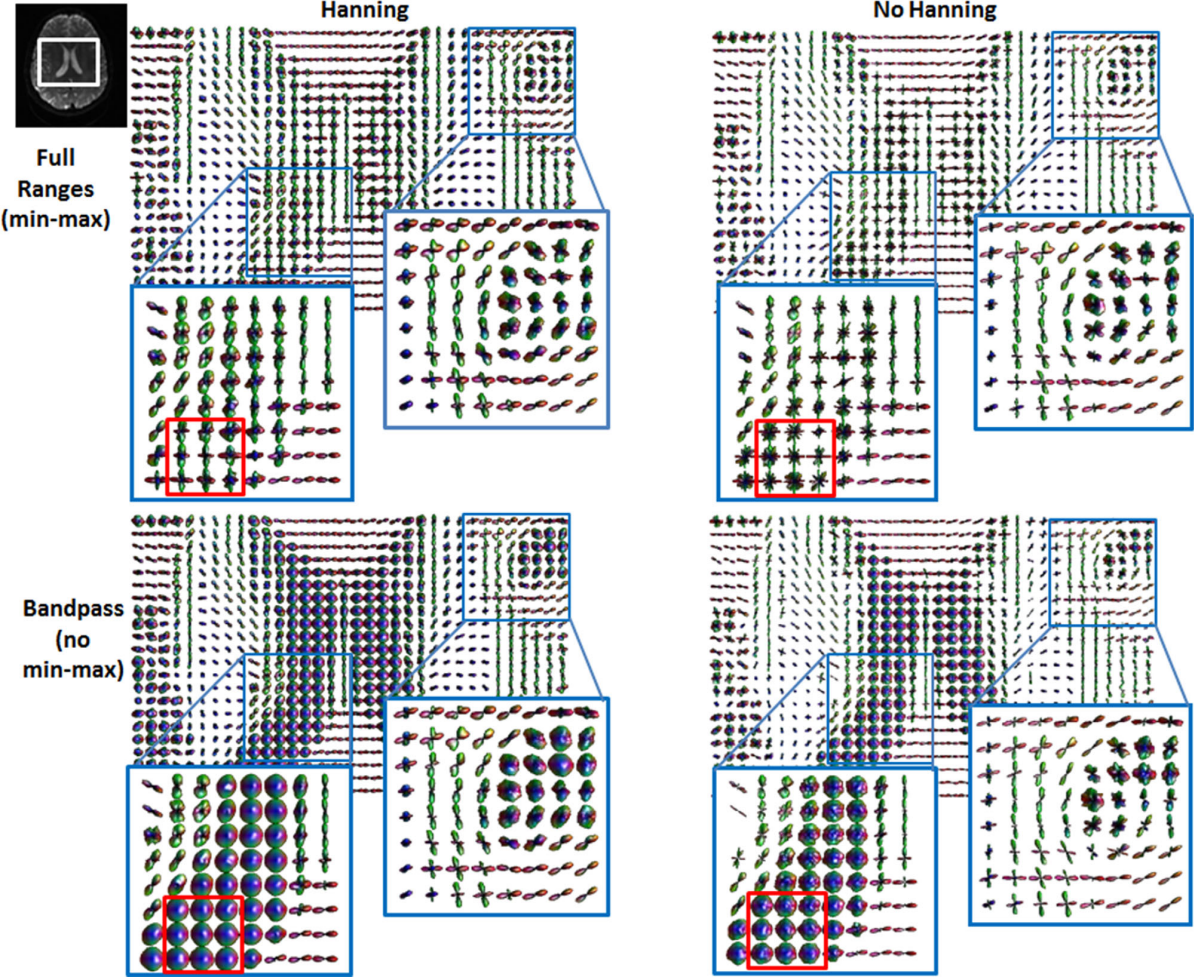
ODF field of a brain region including the corpus callosum and CSF calculated with/without Hanning filtering before diffusion propagator estimation and for full‐range ODF and bandpass versions. Full‐range ODFs were applied min‐max normalization for improved angular resolution.

Figure 7 displays a comparison of different processing steps in the reconstruction of the ODF, on in vivo data, in a region where the contamination by partial volume effects can be observed, using the ranges determined in the simulations.

Figure 7 clearly shows that the use of min–max ODFs obtained either with or without Hanning filter, gives rise to artifactual and regularly structured fiber orientations in the ventricles (red box) where only CSF is present. By using the band‐pass approach, voxels in CSF regions correctly display an isotropic profile. At the same time, crossings in WM regions are better resolved as shown in the selected region of the brain (top right corner).

**Figure 8:**
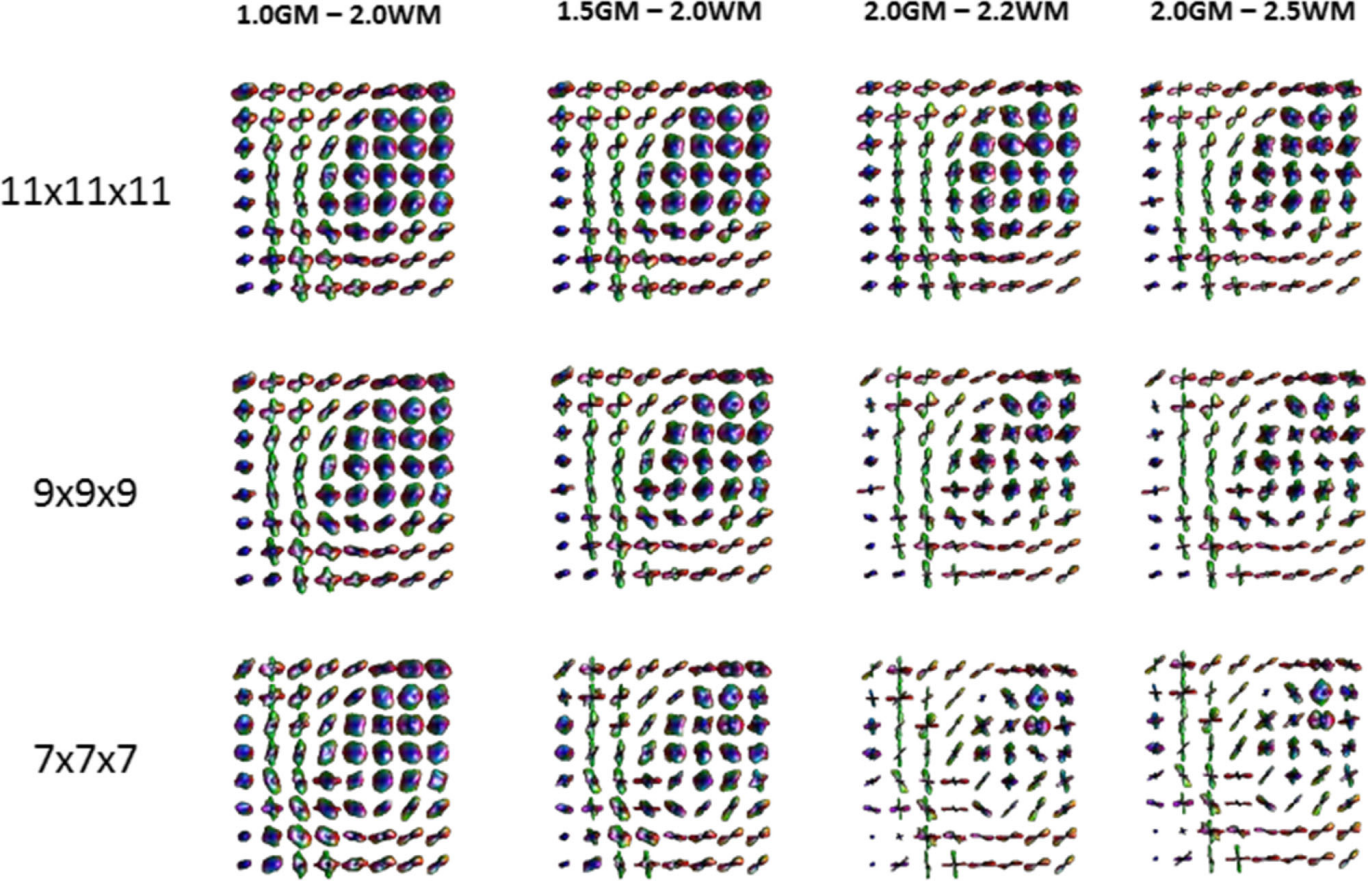
Effect of different integration ranges and grid sizes on the reconstruction of the final ODF. For grid sizes of 11, 9, and 7 points, four different integration ranges were computed and displayed: 1.0 × $R_{\text{GM}}\;$ to 2.0 × $R_{\text{WM}}$; 1.5 × $R_{\text{GM}}\;$ to 2.0 × $R_{\text{WM}}$; 2.0 × $R_{\text{GM}}\;$ to 2.2 × $R_{\text{WM}}$; and 2.5 × $R_{\text{GM}}\;$ to 2.0 × $R_{\text{WM}}$.

Finally, Figure 8 shows how different integration ranges and grid sizes influence the ODF reconstruction for the same brain region as in Figure 7. As expected by moving the integration ranges to higher diffusivity values we observe an increase of angular resolution in the recovered ODF. However, for the smaller sampling schemes the stability of the estimated ODF is reduced precluding a complete recovery of the underlying WM organization.

## DISCUSSION

### Fast Diffusion Artifacts

The main objective of this study was to investigate how stable DSI reconstructions are, when realistic changes occur in the biophysical system. We simulated isotropic, single fiber scenarios, and crossing fiber configurations varying the diffusivity of the system and also the DSI acquisition schemes used to measure it. The expected profile for a diffusion propagator within a fully isotropic voxel is a sphere, and that was the case for the asymptotic acquisition scheme, where the amplitude of the ODF is constant for all isotropic diffusivities tested (Fig. 3). However, for the “state of the art” (i.e., b = 21,000 s.mm^−2^) and “high resolution” (i.e., b = 8000 s.mm^−2^) DSI acquisition schemes, the amplitude of the ODF is not constant and changes along different directions, particularly for higher diffusivity values. This effect is further amplified and can be seen at all diffusivities, for the “medium resolution” (i.e., b = 4000 s.mm^−2^) acquisition scheme. This effect occurs on the faces of the sampled Cartesian grid, where fast diffusion components go beyond the sampled field of view of the propagator inducing aliasing. The consequence is an apparent higher probability displacement along the main axes of the Cartesian grid. This has an effect on the computation of the final ODF profile and can get further amplified when using min–max normalization. Higher diffusivities lead to a more pronounced effect, which may, therefore, be particularly problematic in voxels containing CSF such as the one displayed in the in vivo data in Figure 7 or edema, in pathological tissue.

Even though it is possible to mask out CSF or a lesion for tractography purposes, the presence of voxels with partial volume contaminations may still lead to inconsistent reconstructions 38, 39. The limit at which such aliasing is detected depends on the acquisition parameters and to avoid artifacts, it is necessary to apply a maximum q‐value corresponding at least to twice the maximum displacement present in the diffusion propagator. This gives already good indication in terms of the required sampling density, which must be sufficient to explore that maximum displacement and should be used as basis for the optimization of DSI acquisition schemes 40, 41. This becomes particularly relevant as new hardware capable of achieving higher performances are available today 10, 42.

When we extended this analysis from the isotropic to a single fiber scenario, we found similar effects for all acquisition schemes. Again, only for the asymptotic scenario, the maximum amplitude of the ODF is constant for all diffusivities (Figure 4, left column). We can also see that by only reducing the sampling density from the asymptotic it again leads to significant differences in the amplitude of the ODF. For the “medium” and “high resolution” acquisition schemes, together with changes in the ODF amplitude, additional artifacts not present in the isotropic scenario were also visible, such as the presence of artificial peaks. We further noticed that for the latter two DSI schemes, the presented ODF profile was also asymmetric, which might be attributed to the effect of the Cartesian sampling and residual aliasing effects 15, 43 that add asymmetric features on the final propagator. When we considered the application of Hanning filtering in the ODF reconstruction, some of these artifacts were reduced at the expense of a severe reduction of angular resolution of the ODF. This was even more evident for a crossing fiber scenario, as depicted in a simulation (45 degree crossing) in Figure 5. Here the use of the band‐pass approach to generate the ODF also contributed to further remove some of these artifacts. Finally, the implementation of a threshold in the propagator to exclude low values and ringing effects before ODF reconstruction, also removed remaining spurious peaks present 44.

### Selecting the Right Diffusion Ranges

In the second part of the study, we explored the effect of restricting the integration ranges to specifically probe “axial like” diffusivity profiles (Fig. 2) and in an attempt to minimize some of the problems described. Our method relies on the restriction of the radial integration of the propagator to diffusion ranges that are within expected biophysical meaningful displacements. As it can be seen in Figure 5, this not only allows removing some artifacts from partial volume contaminations but also provides a better angular resolution of the ODF. To further explore the gain in angular resolution, we generated ODFs with different integration bands and compared them with ODFs generated using traditional min–max normalization, with and without Hanning filtering, for pure WM crossings. Figure 6 displays the result of changing both the lower and upper bounds of integration and the best heuristically estimated range appears to be from *α =* $2.5*r_{GM}$ to *β =* $2.5*r_{WM}$. Moreover, for this specific range and for the case where no Hanning filtering is applied (Table 2), the recovered angular resolution is sufficient to resolve a 30‐degree crossing, attaining similar performance of model‐based approaches 30, 36.

This range was further evaluated in real data, and demonstrated an increase of angular resolution of the ODF even with the use of Hanning filtering (Fig. 7). In the top row of Figure 7, we can clearly see the aliasing effect of fast diffusion like components in combination with min–max normalization, as regularly structured crossing fibers can be detected in the ventricles, where an isotropic diffusion profile is expected. On the bottom row, the results of the ODF computed with biophysical integration ranges are shown and these artifacts are removed, even in the absence of Hanning filtering. This can be further appreciated in Figure 7, where a WM region is highlighted and shows higher angular resolution for single and crossing fibers, while no directional information is found in voxels containing CSF, which actually display a spherical profile. To better appreciate the effect of applying the band‐pass approach, real data processed with different integration ranges are also displayed in Figure 8. Additionally, those ranges were applied on two under‐sampled versions of the 11 × 11 × 11 grid dataset. The under‐sampled datasets showed smoother profiles and decreased angular resolution. Even when using the band pass approach it was not possible to significantly improve resolution without losing the underlying WM structural organization.

Finally, it should be noted that band pass approach can also be applied to other model free diffusion techniques, preserving the quantitative nature of the ODF and, therefore, enabling comparisons that are not possible when min–max normalization is used 42. Future work will focus on the optimization of the integration range used.

## CONCLUSIONS

Current diffusion imaging studies are limited by hardware and time constraints, which hinder the use of otherwise very promising techniques in clinical settings 10, 45. In the current study, we have demonstrated the limitations affecting most of the current implementations of DSI. While advances in hardware are likely to help minimize many of the current problems such as low SNR, long scan times, and motion artifacts, other issues are likely to remain; in particular we have identified that, for typical acquisition parameters, fast diffusion components are not well characterized and can lead to aliasing on the diffusion propagator. As a result of this, in pathological tissue, or in voxels contaminated with CSF, the processing methods normally used with DSI may lead to the reconstruction of artifactual fibers when the resulting ODFs are used for tractography.

To tackle this issue, we have introduced a new way of computing the ODF, in a band‐pass manner, which relies on restricting the integration ranges of the propagator based on the expected biophysical displacement of water molecules in the tissue of interest. We have shown that, if the appropriate ranges are chosen, the angular resolution that we obtain for the ODF is comparable (or even superior) to standard ODF processing, and our method has the additional advantage of retaining a quantitative nature of the ODF and can be generalized to other model‐free diffusion imaging techniques.
